# Effect of light needle in the treatment of opioid use disorder: A protocol for a randomized controlled trial

**DOI:** 10.1097/MD.0000000000031451

**Published:** 2022-12-16

**Authors:** Tsuo-Cheng Lu, Chun-En Kuo, Szu-Ying Wu, Yi-Hsun Tsai, Yu-Chiang Hung, Wen-Long Hu, Meng-Chang Tsai

**Affiliations:** a Department of Chinese Medicine, Kaohsiung Chang Gung Memorial Hospital and Chang Gung University College of Medicine, Kaohsiung, Taiwan; b Department of Leisure and Sports Management, Cheng Shiu University, Kaohsiung, Taiwan; c School of Chinese Medicine for Post Baccalaureate I-Shou University, College of Medicine, Kaohsiung, Taiwan; d Fooyin University College of Nursing, Kaohsiung, Taiwan; e Kaohsiung Medical University College of Medicine, Kaohsiung, Taiwan; f Department of Psychiatry, Kaohsiung Chang Gung Memorial Hospital and Chang Gung University College of Medicine, Kaohsiung, Taiwan.

**Keywords:** heroin addiction, laser acupuncture, Light needle, methadone maintenance treatment, opioid use disorder, traditional Chinese medicine

## Abstract

Opioid use disorder occurs worldwide and creates an increasing economic burden and public health crisis. Some problems are associated with using opioid agonists; therefore, there is a need to develop non-opioid treatments to improve acute and long-term opioid withdrawal syndromes.

**Methods:**

We will enroll 100 participants with opioid use disorders receiving methadone maintenance treatment at an addiction treatment center and randomly allocate them to an experimental or control group. The experimental group will receive 12 sessions of light needle therapy within 4 weeks, while the control group will receive sham light needle treatment without any laser output. Urinary morphine levels were assessed before and after treatment. Participants will be asked to self-report their number of episodes or days of heroin use and heroin craving/refusal to use heroin in the previous week before and after treatment on a visual analogue scale score of 0 to 10. Quality of life will be reported using the Short Form-12v2 before and after 4 weeks of treatment. Pulse diagnosis and heart rate variability will be evaluated before and after treatment. Baseline patient characteristics will be compared between the groups using the independent *t* test and the *χ*^2^ test. Data between the 2 groups will be compared using generalized estimation equations, and paired *t* tests.

**Objective:**

This study aims to investigate the effect of adjuvant light needle therapy in patients with opioid use disorder on methadone maintenance treatment.

## 1. Introduction

Drug use disorders occur worldwide and pose an increasing economic burden and a public health crisis. In 2017, an estimated 5.5% of the global population aged 15 to 64 years (a 30% increase over 2009) used illicit drugs in the previous year.^[1]^ Among other illicit drugs, opioid use disorder (OUD) is a common global problem that leads to accidental deaths due to opioid overdoses, newborn withdrawal symptoms due to mothers’ opioid use during pregnancy, potential health sequelae of drug-use behaviors, and huge social and medical expenditures.^[2–5]^ The mechanisms of OUD include the development of dependence, addiction, and biopsychosocial disorders, with risk factors such as sex, country, education, social background, conduct problems in childhood or early adolescence, and opioid availability.^[4]^ Drug addiction is a chaotic entanglement of neurobehavioral and neurobiological factors. It combines impulsion with compulsion based on the most well-known reward system, the mesocorticolimbic dopamine circuit, which contains domains of the hippocampus, prefrontal cortex, ventral tegmental area, and nucleus accumbens.^[6]^ Recent evidence shows that chronic drug consumption reduces the effects of antioxidant and anti-inflammatory synthetic substances in the brain, antioxidant and anti-inflammatory biomolecules secreted by mesenchymal stem cells, and anti-inflammatory microRNAs and anti-miRNAs, which alleviate neuroinflammation and drug cravings.^[7]^ Other studies have indicated that oxytocin signaling plays an important role in drug addiction.^[8]^ The reduction or abrupt cessation of illicit drugs can lead to withdrawal syndrome, which is associated with the impairment of β-endorphin neurotransmission in the arcuate nucleus. This projects to the nucleus accumbens (NAc) of the hypothalamus, eventually leading to physical, mental, and social problems.^[9]^ Currently, the main treatment strategy for OUD is oral opioid substitution treatment (OST). The World Health Organization (WHO) guidelines recommend the administration of methadone, buprenorphine, slow-release oral morphine, and levomethadone to treat opioid dependence.^[10]^ Methadone is a long-term opioid substitute that has been widely accepted and well-studied worldwide since 1965.^[11]^ The advantages of methadone treatment include blocking euphoric effects, reducing the frequency of opioid use and drug cravings, and decreasing the mortality rate and the spread of infectious diseases.^[12]^ Owing to the contribution of methadone substitution therapy, opioid use is the most common form of addiction.^[13]^ Despite remarkable improvements in the knowledge of the biochemical mechanisms of drug addiction, therapeutic options are relatively deficient and confined, with poor compliance and dangerous side effects.^[8,14]^

Acupuncture based on the principles of the traditional Chinese meridian theory is performed by inserting fine, sterile needles into acupuncture points to achieve “Qi and blood,” or a “yin and yang” state of harmony.^[15]^ Acupuncture therapy is inexpensive and simple, has fewer side effects than other treatments used for opiate addiction, and is safe for both pregnant and parturient women.^[16,17]^ The application of acupuncture to opiate addiction was 1^st^ reported by Dr Wen in Hong Kong in 1972, who reported that electroacupuncture at 4 body acupoints and 2 ear acupoints could alleviate opioid withdrawal symptoms in patients with opiate addiction.^[18]^ A breakthrough protocol was developed in 1985 by Dr M. Smith, the head of the US National Acupuncture Detoxification Association (NADA). The NADA protocol reported that the insertion of 5 needles into bilateral ear acupoints at the sympathetic, shenmen, kidney, lung, and liver points could relieve withdrawal symptoms, prevent drug cravings, and increase patient compliance in long-term therapeutic programs.^[19]^ In 1996, the WHO accepted acupuncture as a treatment for drug abuse.^[20]^ This method of healing has been reported to normalize hyperreactivity or hypoactivity of the mesolimbic dopamine system, activate analgesic effects via both the μ and κ receptors, prevent relapse of drug-seeking behavior by regulating neurotransmitters, and alleviate illicit drug withdrawal syndrome, including physical and psychological symptoms.^[21–24]^

Light needle or laser acupuncture (also termed low-level laser therapy), the use of nonthermal, low-intensity laser beams that penetrate tissues and stimulate acupoints, has recently become more popular among acupuncture practitioners owing to its noninvasive, safer, pain-free, time-saving properties, lower manual skill requirement, and fewer adverse effects than traditional acupuncture.^[25]^ A study showed that light needles achieve neural responses similar to those of needle acupuncture.^[26]^ These features make light needles a more acceptable and effective option for patients on an abstinence plan. A previous study suggested that laser intervention was beneficial for opioid addicts to address drug cravings and improve their quality of life.^[27]^ This study aims to investigate whether light needle treatment combined with methadone maintenance treatment (MMT) is effective in the management of OUD.

## 2. Methods

### 2.1. Ethics approval

The trial was approved by the Chang Gung Medical Foundation Institutional Review Board (IRB no. 202200041A3). The protocol was registered with ClinicalTrials.gov (identifier NCT05341219) and Chinese Clinical Trial Registry (identifier ChiCTR2200059497). Written informed consent will be obtained from each participant. Personal information about the potential and enrolled participants will be collected, shared, and maintained in a separate closet to protect confidentiality before, during, and after the trial.

### 2.2. Study design

**Figure 1.**
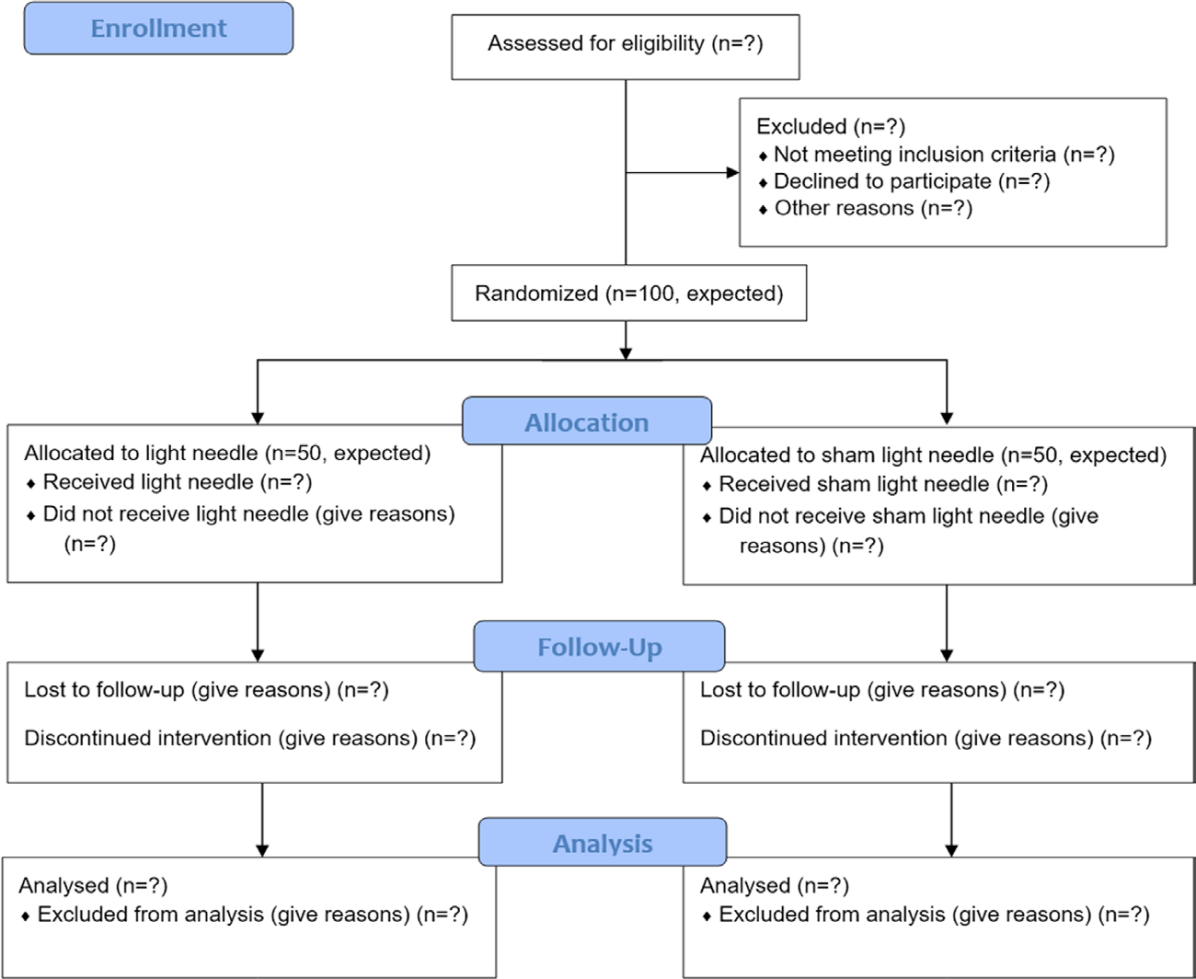
Flowchart showing movement of patients through the study.

This randomized controlled trial is being conducted in the Department of Psychiatry and Chinese Medicine at Kaohsiung Chang Gung Memorial Hospital. The study was initiated in March 2022 and will continue until February 2024. Patients enrolled from our institution’s addiction treatment center are being randomly allocated to the experimental group (light needle plus methadone maintenance treatment, n = 50, expected) or the control group (light needle without laser output plus methadone maintenance treatment, n = 50, expected). The study participants will receive 12 sessions of light needle therapy within 4 weeks. The study design is illustrated in Figure 1.

### 2.3. Participants

The diagnosis of OUD will be confirmed using the diagnostic criteria of the Diagnostic and Statistical Manual of Mental Disorders, 5^th^ Edition. We will recruit patients aged 20 to 70 years with OUD who have received methadone maintenance treatment for at least 1 month and have provided informed consent. A psychiatrist will assess the eligibility of each potential participant before recruitment. Patients with critical illness, those who have taken Chinese medicine or received acupuncture within 30 days, those who are considered unsuitable for recruitment by a physician, and those who are not willing to provide informed consent will be excluded.

### 2.4. Sample size and randomization

We calculated a sample size of 98 in accordance with a 2-way repeated-measures analysis of variance with a medium effect size of 0.25, pre- and post-measurements for each of the 2 groups, a significance level (*α*) of 0.05, and a desired power (1–*β*) of 0.80.^[28]^ Expecting a 2% dropout rate, 100 participants will be recruited. A random permutation block of block size 4 is selected using a research randomizer that generated a random sequence.^[29]^ Allocation concealment was performed by using numbered containers. The researchers recruited and assessed participants in sequentially numbered, opaque, sealed, and stapled envelopes. Trial participants, outcome assessors, and data analysts will be blinded after assignment to interventions labeled A and B for both groups.

### 2.5. Interventions

**Figure 2.**
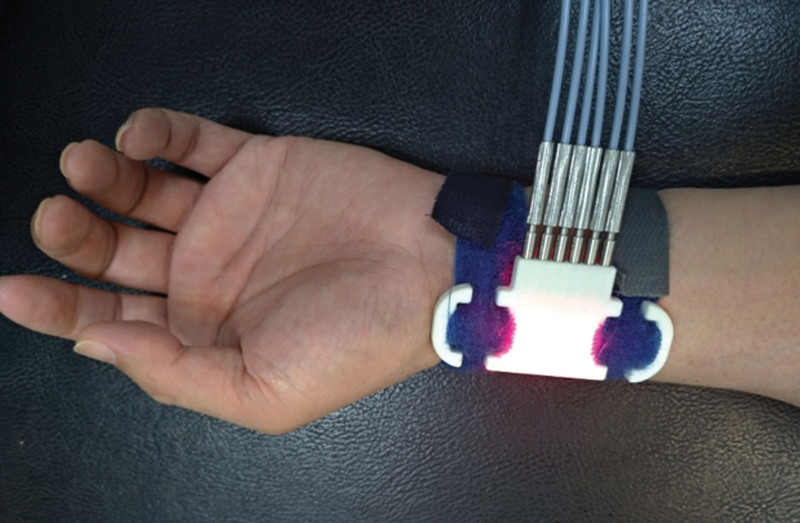
Light needle therapy between LU7 and LU9.

The study participants will receive 12 sessions of light needle therapy within 4 weeks using a gallium aluminum arsenide laser (Physiolaser Olympic; maximal power, 60 mW; wavelength, 655 nm; area of probe, 0.008 cm^2^; power density, 7.5 W/cm^2^; pulsed-wave; 3305 Hz, 1168 Hz; RJ-Laser, Reimers & Janssen GmbH, Waldkirch, Germany). Similar acupuncture points will be used in both groups. Participants in the control group will receive sham light needle therapy without laser stimulation, whereas those in the experimental group will receive 135 J of energy delivered from 6 light needles placed between LU7 and LU9 (Fig. 2). Light needle therapy was applied at each point for 15 minutes. The same trained and experienced physician will perform laser application for each participant. The latter is a licensed traditional Chinese medicine physician in Taiwan with > 10 years of traditional acupuncture experience and > 5 years of laser acupuncture experience. The physician and participant will use protective goggles and a laser shield, respectively, to inhibit visual perception during the light needle treatment.

### 2.6. Outcome measurements

Outcome measurements will consist of subjective reporting of heroin use, quality of life, and objective urinary morphine levels. The primary outcomes are urinary morphine levels and self-reported times or days of heroin use during the previous week before and after the 4-week intervention period. The secondary outcomes are self-reported visual analogue scale (VAS) scores (0–10) for heroin craving/refusal to use heroin in the previous week and quality of life using the Short Form-12v2® (SF-12v2) before and after a 4-week intervention. The participant’s pulse diagnosis and heart rate variability before and after 4 weeks of intervention will be documented.

A VAS score of 0 for heroin craving indicates no heroin craving, while a score of 10 indicates the strongest craving. A VAS score of 0 for refusal to use heroin means no refusal, whereas a score of 10 indicates complete refusal. The SF-12v2 Health Survey is a multipurpose, short-form health tool consisting of 12 questions that generates an 8-domain overview of functional health and well-being (Physical Functioning, Role-Physical, Bodily Pain, General Health, Vitality, Social Functioning, Role-Emotional, Mental Health), 2 psychometric-based summative measures of physical and mental health, and a preference-based health utility index. PRO CoRE is part of the Smart Measurement® System product suite and QualityMetric’s upgrade to QualityMetric Health Outcomes™ scoring software, which will be used to score the SF-12v2 Health Survey.

Reasons for participants not completing follow-up or withdrawing from the study include adverse events/comorbidities, poor response to treatment, failure to return to follow-up, failure to meet selection criteria at enrollment, other breaches of the protocol, and denial of treatment. These will be documented as potential adverse events.

### 2.7. Statistical analysis

Data will be displayed as mean ± standard deviation. Baseline participant characteristics between the experimental and control groups will be assessed and compared using independent *t* tests and chi-square tests. The differences between the 2 groups will be compared using an independent *t* test. Paired *t* test will be used to compare the 2 groups. All analyses will be performed using the Statistical Package for the Social Sciences (SPSS) for Windows version 22 (Statistics 22, IBM Corp., Armonk, NY). Statistical significance is set at P < .05.

### 2.8. Data monitoring

A data monitoring committee (DMC) is not required, as light needle therapy is a routine and noninvasive intervention.

### 2.9. Protocol

This article follows the principles of the Standard Protocol Items: Recommendations for Interventional Trials (SPIRIT) Guidelines.^[30]^

## 3. Discussion

The well-known mechanism of acupuncture in opiate addiction treatment is the activation of endogenous opiates and neurotransmitters, such as endorphins, enkephalins, endomorphins, dynorphins, epinephrine, serotonin, norepinephrine, dopamine, and amino acids, including GABA, in the nervous system.^[15,22,31]^ Studies showed that acupuncture modulates mesolimbic dopamine release, thus reducing withdrawal symptoms and suppressing the reinforcing effects on drug-seeking behavior by restoring endogenous dopamine levels in the NAc.^[21,32,33]^ Acupuncture can provoke β-endorphin and enhance DA release in the NAc.^[34]^ As more possible acupuncture mechanisms have been revealed, more methods for stimulating acupuncture points have been developed, including emitting laser light onto the acupoints.^[35]^

Light needles possess the advantages of traditional acupuncture and photobiological effects by releasing photon energy into the target tissue.^[36]^ Laser wavelengths absorbed by mitochondrial cytochrome C oxidase protein, associated with ATP processing, have the effects of activating mitochondrial activity, stimulating RNA and DNA synthesis, stabilizing cell membranes, increasing metabolism, modulating enzymatic activity, and restoring multiple body functions.^[37,38]^ In addition, light needles improve impaired cell conditions as cells undergo oxidative stress by increasing reactive oxygen species and dissociating nitric oxide from cytochrome C oxidase, which increases the overall cell redox potential and enhances superoxide dismutase and catalase activity.^[39]^ Elevation of redox power activates various intracellular signaling pathways, including the synthesis of nucleic acids, proteins, and enzymes, and the initiation of cell cycles.^[40]^ Light needle also regulate gene expression, which enhances cell growth and inhibits apoptosis.^[38]^ The cellular effects induced by light needles reflect their ability to remodel long-term changes in cells and their benefits for OUD-induced oxidative stress and inflammation.^[41,42]^

According to the traditional Chinese medicine theory, patients with OUD experience excessive blood stasis, weak qi and blood, deficiency of righteous qi, and disorder of visceral qi movement, all of which can affect the brain and collateral organs, resulting in mental abnormalities and influenced cognition.^[43]^ The lungs govern qi and help the heart vessels to circulate nourishing blood. LU9 is located in the cunkou, and all blood vessels converge in the lungs, meeting at LU9. This is where the qi of zang-fu vessels converges, where they can regulate qi and blood and dredge the blood vessels.^[44]^ Overall, we expect that a combination of light needles with MMT will create a positive synergistic effect on reducing drug cravings, relieving adverse effects, elevating quality of life, and strengthening health physically and psychologically, as previous findings have shown that acupuncture is effective for OUD.

## Acknowledgments

The authors thank the Biostatistics Center at Kaohsiung Chang Gung Memorial Hospital for developing the statistical analysis protocol that will be used in this research.

## Author contributions

**Conceptualization:** Wen-Long Hu, Meng-Chang Tsai.

**Data curation:** Wen-Long Hu.

**Formal analysis:** Wen-Long Hu.

**Funding acquisition:** Wen-Long Hu.

**Investigation:** Tsuo-Cheng Lu, Chun-En Kuo, Szu-Ying Wu, Yi-Hsun Tsai, Wen-Long Hu, Meng-Chang Tsai.

**Methodology:** Wen-Long Hu, Meng-Chang Tsai.

**Project administration:** Wen-Long Hu.

**Resources:** Meng-Chang Tsai.

**Software:** Wen-Long Hu.

**Supervision:** Wen-Long Hu, Meng-Chang Tsai.

**Validation:** Chun-En Kuo, Szu-Ying Wu, Yi-Hsun Tsai, Yu-Chiang Hung, Wen-Long Hu, Meng-Chang Tsai.

**Visualization:** Yu-Chiang Hung.

**Writing – original draft:** Tsuo-Cheng Lu, Wen-Long Hu.

**Writing – review & editing:** Yu-Chiang Hung, Meng-Chang Tsai.
